# Epigenomics in COVID-19; the link between DNA methylation, histone modifications and SARS-CoV-2 infection

**DOI:** 10.2217/epi-2021-0057

**Published:** 2021-04-20

**Authors:** Milad Shirvaliloo

**Affiliations:** ^1^Student Research Committee, Tabriz University of Medical Sciences, 5166 Tabriz, Iran; ^2^Faculty of Medicine, Tabriz University of Medical Sciences, 15731 Tabriz, Iran

**Keywords:** COVID-19, DNAm, epigenetic mechanisms, epigenomics, histone modifications, SARS-CoV-2

Epigenomics is the collective study of all epigenetic modifications that can affect the genome, the most pivotal of which are DNA methylation (DNAm), histone modifications (e.g., acetylation, citrullination and phosphorylation) and nucleosome remodeling. The latter refers to the dynamic reorganization in the spatial arrangement of chromatin by means of repositioning the nucleosomes, which are short segments of DNA, consisting of approximately 200 base pairs, coiled around eight histone proteins. The resulting alterations in the structure of these bead-like complexes may favor or oppose transcription, determining the course of disease pathogenesis [1], such as coronavirus disease 2019 (COVID-19). Known to correlate inversely with gene expression, DNAm might be associated with increased susceptibility of the pulmonary tissue to COVID-19 [2] and a higher predisposition to this viral disease in patients with pancreatic [3] and prostate cancer [4]. A lesser known epigenetic mechanism, ‘X inactivation’ is thought to be connected with the function of innate immunity against COVID-19, particularly in male patients [5]. Furthermore, immunity is also influenced by the extensively diverse histone modifications, especially histone citrullination [6]. Nucleosome remodeling, mediated by contextually important proteins such as SMARCA4 and SMAD3, is another epigenetic mechanism that can affect the course of disease, assuming a patient-wise unfavorable role in the case of COVID-19 [7]. The footprint of epigenomics can even be traced back to prenatal life, when developmentally crucial genes such as *PEG10* and *ECE1*, can be affected by DNAm in response to maternal COVID-19 [8], indicating the overall significance of epigenetics in the recent viral pandemic.

## COVID-19: unwelcome infection facilitated by epigenetic modifications

A consequence of SARS coronavirus 2 (SARS-CoV-2) infection, COVID-19 is perhaps the greatest health issue of the century, affecting more than 45 million individuals, with a global death toll exceeding one million [7]. The recent, less-anticipated advent of novel SARS-CoV-2 variants with altered Spike proteins, including the strain with ‘Spike N453Y’ mutation reported in Netherlands and Denmark; and the more overwhelming variants with ‘Spike D614G’ and ‘N501Y’ mutations spreading in England, has caused concerns regarding the efficacy of the recently approved vaccines in the future. The N501Y variant, in particular, has been suggested to be 56% more transmissible, owing to the enhanced binding affinity of the Spike protein to its polypeptide receptor, ACE2, on the host cell [9]; a finding with potential epigenetic implications.

Containing a positive-sense ssRNA, SARS-CoV-2 is an enveloped β-coronavirus coated with Spike protein on its surface, which relies on the interaction between this protein and the transmembrane ACE2 on the host cell for viral entry. The epigenetic modifications, especially DNAm, regulating the expression of ACE2 had been known well before the emergence of SARS-CoV-2 [2].

Nonetheless, the rising prominence of epigenomics in the expansive research field of COVID-19 is not solely due to the role of ACE2 in the pathophysiology of the disease, as 332 human proteins have recently been identified to interact with SARS-CoV-2 proteins in several ways. A noteworthy example is HDAC2, which is involved in the initiation of immune responses against viruses [10]. HDAC2 contains a cleavage site that can be targeted by NSP5, a nonstructural protein belonging to SARS-CoV-2. Interaction with NSP5 may lead to inhibition of HDAC2, perturbing the nuclear localization of this enzyme. This is a consequential phenomenon, since HDAC2 catalyzes deacetylation of H4K16ac; an unusual epigenetic modification to histone H4 at the 16th lysine residue, which can either activate or repress transcription [2]. Thus, interactions between SARS-CoV-2 and the host cell may alter epigenomics in unpredictable ways.

## DNAm: major epigenetic mechanism responsible for enhanced viral entry

A typically transcription-repressing epigenetic modification, DNAm occurs when the adenine or cytosine base in a nucleotide is bound to methyl groups by the action of DNA methyltransferases. Cytosine is widely methylated at position C5 in mammalian cells, hence the term, C5-methylcytosine (5-mC). This methylated type of cytosine usually occurs in pairs of CpGs, which are specific sites in the DNA where a cytosine nucleotide is immediately followed by a guanosine nucleotide in the 5′–3′ direction. Cytosine is methylated in approximately 80–90% of CpGs. The remaining 10–20% of unmethylated CpGs can usually be found in high concentrations at specific regions of DNA known as ‘CpG islands’, which are frequently located at gene promoters [11].

CpG methylation is inversely correlated with gene expression; high levels of methylation at promoter-associated CpGs result in low levels of gene expression. Accordingly, one reason for the high expression of ACE2 in the lungs, liver and brain, lies within the sparsely methylated CpG islands at the promoter of ACE2 in these organs, rendering them more vulnerable to SARS-CoV-2-induced damage [2]. This hypothesis has been further supported by a recent study concluding that the footprint of epigenetic modifications is present in various stages of SARS-CoV-2 pathogenesis, particularly cell membrane-virus fusion [12].

## Altered DNAm of CTSL/B & OR51E2 in cancer; increased vulnerability to COVID-19

Beside affecting the pathogenesis of SARS-CoV-2 in healthy individuals, DNAm may also have important implications in the case of individuals with pre-existing malignancies. Cancer is a well-known risk factor associated with increased vulnerability to COVID-19. According to the COVID-19 and Cancer Consortium (CCC19), the highest incidence of SARS-CoV-2 infection among individuals with cancer is mostly observed in patients with hematological, breast, gastrointestinal and thoracic malignancies, reported as comorbidities in 65 percent of COVID-19 diagnoses in populations affected by cancer [3].

Pancreatic adenocarcinoma (a gastrointestinal malignancy) has recently been found to coincide with decreased DNAm of CTSL/B; two cysteine proteases involved in the lysosomal catabolism of proteins. These particular proteases are crucial to SARS-CoV-2 pathogenesis, as they cleave the viral Spike protein into the S1 and S2 subunits, further mediating the entry of the virus into the host cell. It has been suggested that hypomethylation of CTSL/B in pancreatic adenocarcinoma may lead to overexpression of CTSL/B, resulting in greater susceptibility to COVID-19 [3]. The clinical significance of this finding is that cathepsins can be selectively inhibited by certain therapeutic agents, such as calpain inhibitor III, that targets CTSL/B. Reportedly, inactivation of CTSL/B is correlated with diminished replication of SARS-CoV-2, opposing the virus-induced cell death [7].

The fifth leading cause of mortality in the world, prostate cancer, particularly prostate adenocarcinoma, is suspected to be correlated with COVID-19 in several ways. In 2020, an investigation by Montopoli *et al.* revealed a potential inverse link between androgen-deprivation therapy and vulnerability to COVID-19, suggesting the protective role of androgen-deprivation therapy against SARS-CoV-2 infection in patients with prostate cancer [13]. Hence, androgen receptors were speculated to be involved in the pathogenesis of SARS-CoV-2, since activation of these receptors is believed to result in the upregulation of TMPRSS2; a transmembrane protease facilitating the entry of SARS-CoV-2 into the host cell [14].

In addition to androgen receptors, their olfactory counterparts have also been found to constitute a crosstalk between prostate cancer and COVID-19. Olfactory receptors can often become compromised following SARS-CoV-2 infection, resulting in impairment of the sense of smell or hyposmia. An olfactory receptor, OR51E2 is also expressed in the smooth muscle cells residing in the airways. Pathological increase in the mass of smooth muscle in the airways, recognized as a hallmark of asthma, may occur due to potential overexpression of OR51E2. Interestingly, the promoter of OR51E2 was reported to be hypomethylated in prostate adenocarcinoma, which is correlated with an elevated expression of this receptor. Thus, owing to a common epigenetic modification, prostate cancer could possibly be considered a risk factor for severe COVID-19 [4].

## Trained immunity: chronological innate immunity in the light of epigenetic regulation of the X chromosome

The first line of defense against foreign antigens, the innate immune system is often regarded as a primitive network of less-specialized recognizing and phagocyting cells, that react similarly upon every exposure to any potential pathogen. Assumed exclusive to adaptive immunity, in the contemporary literature, immunological memory is an incontrovertibly important aspect of the immune system, that facilitates rapid recognition and elimination of pathogens on subsequent exposures [15]. However, the concept of ‘trained immunity’ may suggest otherwise. The term was coined to describe the recently elucidated process through which the innate immune system, in following exposures, exhibits an enhanced response to the same foreign material [16], for example, SARS-CoV-2. In a fashion similar to the immunological memory, trained memory is subject to several regulatory processes, particularly epigenetic mechanisms [15].

Innate immunity comprises monocytes, macrophages, natural killer and dendritic cells; each of which are able to modify their prospective behavior based on the body of information collected from past encounters, in other words, trained memory, with the help of an expansive array of receptors [17]. Located on the X chromosome, *TLR7* is a gene coding for TLR7, that mediates type I and II interferon responses. Interferons (IFN) are major cytokines responsible for regulating immune response against viruses. Increased susceptibility to viral infections is an unfavorable outcome associated with conditions disrupting the action of IFN [18], such as COVID-19. Several investigations have suggested that downregulation of this response due to epigenetic mechanisms might lead to a more severe disease in patients with SARS-CoV-2 infection [2].

Known as a mechanism of gene dosage compensation, X inactivation is an epigenetic regulatory process responsible for the silencing of one of the two copies of the sex chromosome in females. X upregulation, in complement, is another epigenetic compensatory mechanism evolved to narrow the genotypic gap between males and females, since men only possess one copy of the X chromosome [19]. Similar to *ACE2*, *TLR7* happens to avoid the silencing effects of X inactivation; a benevolent phenomenon which is speculated to be accompanied by beneficial outcomes, especially in the case of male patients [2]. Not long ago, an investigation led by van der Made reported that loss-of-function mutations in *TLR7* were associated with a more severe disease in male patients infected with SARS-CoV-2, compared with other COVID-19 patients of the same age and sex [5].

## Histone modifications: the perplexing epigenetic circuit behind immune response to SARS-CoV-2

From an epigenetic point of view, IFN are crucial to survival, since they can affect the expression of roughly 10 percent of all human genes, particularly certain sequences, which are collectively termed 'ISG’ which are interferon stimulated. An ISG is basically any gene that can be induced following an interferon response. *IFN-γ*, a type II interferon, is a well-known ISG [20]. A number of studies have recently hypothesized that histone modifications might be involved in the regulation of IFNs and ISGs. In 2010, two investigations reported a high density of histone acetylation and RNAPII, and thus, significant transcriptional activity at certain promoters in activated macrophages and dendritic cells. The primary arm of pathogen recognition, TLRs are usually marked with H3K4me3 [2], a transcription-activating histone modification, denoting trimethylation at the fourth lysine residue of histone H3 [21].

Accordingly, one may conclude that histone modifications reinforce innate immunity by regulating TLRs, and ultimately, ISGs. However, ISGs exhibit a more heterogenous distribution pattern of epigenetic modifications at their promoters, since they also contain transcription-repressing histone modifications, such as H3K9me2 and H3K27me3 and CpG islands, which may not support the speculation that such modifications should act in favor of an antiviral immune response. Besides, additional stimulation from chromatin or nucleosome remodelers is usually required for the transcription of ISGs [2].

## SWI/SNF: chromatin remodeling complex in favor of cell death induced by COVID-19

SWItch/sucrose nonfermentable (SWI/SNF) is a subfamily of ATP-dependent nucleosome remodeling complexes, encompassing several proteins and polypeptides, that regulate gene expression through the rearrangement of nucleosomes, altering chromatin accessibility. A group of proteins known collectively as ‘SWI/SNF-related matrix-associated actin-dependent regulators of chromatin’ are heavily involved in the function of the SWI/SNF complexes. SMARCA4 is one such protein, that has recently been introduced as a gene specific to SARS-CoV lineage, particularly SARS-CoV-2, by a genome-wide CRISPR analysis. The study, led by Wei, has reported a proviral role for SMARCA4, as well as SMAD3; a protein encoded by the *SMAD3* gene, that transmits signals originating in the cell membrane to the nucleus, mediating the TGF-β pathway [7].

These findings suggest the potential involvement of SMARCA4 and SMAD3 in the entry of SARS-CoV-2 into the host cell, and the ultimate virus-induced cell death. However, it should be noted that this analysis was conducted on the Vero-E6 cell line; a type of cell extracted from *Chlorocebus sabaeus* (African green monkey), that does not express IFN type I. Regardless, inhibition of SWI/SNF-associated proteins could be of therapeutic value in the case of COVID-19. The hypothesis was tested with PFI-3, an epigenetic chemical probe and SIS3, which target SMARCA4 and SMAD3, respectively. Promising results were reported for both therapeutic agents, indicating their protective effects against SARS-CoV-2-induced cell death [7].

## NETosis: inappropriate cell death guided by histone citrullination

A morbid factor contributing to organ damage in patients with COVID-19, NETosis is a controlled type of cell death, that serves as a defense mechanism against pathogens. The process, which is essentially a form of innate immunity, relies on entangled trap-like aggregates of DNA and proteins termed ‘neutrophil extracellular traps’ (NETs). Containing histones as well, NETs are the product of regulated neutrophil death, suggested to be guided from epigenetic modifications, particularly citrullination of histone H3. A specific marker of NETs, citrullinated histone H3 (Cit-H3) was found in elevated amounts in blood following SARS-CoV-2 infection [2]. One investigation, by Leppkes *et al.* has suggested a positive correlation between Cit-H3, IL-8 and granulocyte count, indicating that elevation in the level of Cit-H3 is accompanied by a concomitant increase in the production of IL-8 and the count of granulocytes, in other words, neutrophils, eosinophils and basophils [6].

Interestingly, high levels of NETs were also detected in the pulmonary tissue and tracheal secretions of patients affected by COVID-19, which may partly explain the bronchial and alveolar damage observed frequently in these patients. Suspected to be involved in COVID-19-associated thrombotic events, Cit-H3 has reported to be positively correlated with the count of platelets [2], whose activity is implicated to be strengthened in COVID-19 patients due to a 5.5-fold upregulation of PF4, as indicated by a transcriptomics analysis [22]. Hence, inhibition of platelet aggregation, through antiplatelet medications such as dipyridamole, might be a promising strategy to counteract the adverse systemic impact of Cit-H3; a hypothesis which is currently being tested in clinical trials [2].

## Histone citrullination: PADs against transcription-repressing modifications

Citrulline is the product of a family of protein L-arginine-converting enzymes called PADs or PADIs, that are primarily involved in a form of post-translational modification known as ‘arginine deamination’ or citrullination. The family comprises five highly conserved enzymes, namely PAD1-4 and PAD6 [23], which are activated following a surge in the concentration of intracellular calcium. This clinically important finding was suggested by a number of studies that noted citrullination of histone H3 and H4 arginine residues, and subsequent chromatin decondensation upon treatment of human promyelocytic leukemia (HL-60) cells with calcium ionophores increased the permeability of cell membranes to calcium ions [2].

PADs have recently been implied to hold value in the field of epigenetics, since they can positively regulate transcription. Of particular interest is PAD4, that might be a potential inhibitor of H3K9me2. Reported by Wiese *et al.* PAD4 favors transcription through citrullination of HP1γ. The primary reader of H3K9me2, HP1γ is incapacitated upon citrullination, resulting in counter-regulation of H3K9me2 in favor of transcription [24].

Transcription can also be altered by citrullination of RNAPII, especially Rpb1; the largest subunit of this RNA polymerase. An increase in the interaction of citrullinated RNAPII with super elongation complex (SEC), a highly active complex of P-TEFb, was stipulated in 2019 [25]. SEC is of crucial prominence in cellular development and disease pathogenesis, since it prompts rapid transcription of immediate early genes in case of RNAPII promoter-proximal pausing; a regulatory mechanism during early elongation [26]. Promoter-proximal pausing is lifted in response to RNAPII citrullination, resulting in an improved interaction between RNAPII and SEC, and ultimately, enhanced transcription [25].

The significance of histone citrullination in the case of SARS-CoV-2 infection is in that this particular type of epigenetic modification is also associated with malignancies (through the tumor suppressor protein p53), thrombotic events and autoimmune diseases, which increase the vulnerability of individuals for developing a more severe form of COVID-19 [2].

## Prenatal epigenomics: potential link between maternal infection & altered DNAm

In addition to their regulatory functions in adults and children, epigenetic modifications are crucial to normal development of human embryo, since prenatal exposure to maternal stress, triggered by environmental stress, may result in physical, emotional and cognitive defects in the offspring. This is thought to be, in part, due to consequent alterations occurring in the DNAm of specific genes involved in the regulation of stress and cognitive development of the fetus [27].

Published in December 2020, a DNAm analysis on the heart and kidney of murine models with SARS-CoV-2 infection identified more than 200 differentially methylated regions (DMRs) in the genome of both organs, one-tenth of which were associated with two particular genes with prenatal implications. The two genes, *PEG10* and *ECE1*, were demonstrated to be neighbored by multiple DMRs. Located on chromosome 7, *PEG10* is a paternally expressed imprinted gene, containing several DMRs within its first exon. The presence of these DMRs may render *PEG10* subject to epigenetic modifications in the form of unspecified altered DNAm following SARS-CoV-2 infection, albeit, in the murine models of COVID-19. The clinical prominence of this finding is in that the loss of function of *PEG10* can lead to early embryonic death [8].

A proteolytic enzyme encoded by *ECE1* on the first chromosome, endothelin converting enzyme 1 regulates the processing of potent vasoconstrictors, including endothelin 1–3. Unfunctional copies of *ECE1* are correlated with cardiac defects and autonomic dysfunctions in neonates. Several DMRs were also identified, by the same analysis, upstream to the transcription start site of *ECE1*, whose hypermethylation were associated with downregulation of *ECE1* [8]. This might potentially explain the hypotensive state observed frequently in patients with critical COVID-19, necessitating administration of vasopressors [28], however, such speculations need to be confirmed by prospective studies.

## Future perspective

Concerned with genotype-sparing modifications to phenotype, epigenetics is an indispensable aspect of COVID-19 research, which has aided scientists to further appreciate the undisclosed pathologic alterations in the regulation of gene expression upon being exposed to SARS-CoV-2. The two pillars of epigenomics, DNAm and histone modifications have frequently been reported to facilitate or oppose the pathogenicity of SARS-CoV-2 in human cells. In the pressing case of COVID-19, viral entry, immune responses such as NETosis and TGF-β pathway, and certain malignancies are believed to be correlated with epigenetic mechanisms; with several studies exploring the significance of such mechanisms well before birth, on prenatal level. Epigenetic investigations can also be of great help at identification of candidate therapeutic agents, such as PFI-3, SIS3 and calpain Inhibitor III, for a better management of COVID-19, particularly in the case of patients with comorbidities. Although based on documented evidence, further research is warranted to validate the authenticity of the recent discoveries discussed in this commentary.
